# Manual of Diseases of the Ear

**Published:** 1897-06

**Authors:** 


**Manual of Diseases of the Ear.** By THOMAS BARR, M.D. Second Edition. Pp. xxiii., 415. Glasgow: James Maclehose and Sons. 1896.

The second edition of this work is a great improvement on the former one. The size of the page has been increased, the general type is larger, and bold black type has been introduced for paragraph headings. There are three additional chapters, and the number of illustrations has been doubled. Among the latter are thirty-four reproductions of photographs, many of which, especially those depicting dissections of the temporal bone, are very good, and likely to prove useful. The work is neither too bulky for the student nor too meagre for the requirements of the practitioner, but is calculated to meet the wants of both.

Among the improvements in this edition we would especially note the additions to the chapters on purulent disease of the ear and its consequences. Operations on the mastoid process and tympanic attic are now described very fully and well, and good descriptions are given of the symptoms and treatment of vascular complications, such as sinus phlebitis and inflammation of the internal jugular vein. We have no hesitation in recommending this book as one of the best and most practical, as well as most readable, on the subject in the English language.

